# Reprowebs: a conceptual approach to elasticity and change in the global assisted reproduction industry

**DOI:** 10.1057/s41292-021-00260-6

**Published:** 2021-10-09

**Authors:** Anika König, Heather Jacobson

**Affiliations:** 1 Institute of Social and Cultural Anthropology, Freie Universität Berlin, Landoltweg 9-11, 14195 Berlin, Germany; 2 Department of Sociology & Anthropology, University of Texas at Arlington, Arlington, TX USA

**Keywords:** Global reproductive industry, Assisted reproductive technologies, Reproductive travel, Reprowebs, Meshwork, Gestational surrogacy

## Abstract

In the last few decades, assisted reproductive technologies (ARTs) have become increasingly transregional and transnational, often involving travel within or between countries or even continents. Until recently, the global ART industry was marked by so-called ‘reprohubs’—places (such as southern California, Dubai, Anand, and Mumbai) specializing in the provision of reproductive services. While reprohubs continue to exist, in the last few years, many have splayed out, transforming into something more akin to webs that encompass, but go beyond these hubs. These webs show a unique dynamic capability to tighten, entangle, or extend in reaction to local and global changes, a characteristic which became particularly obvious during the global Covid-19 pandemic. In this paper, we propose conceptualizing this new dynamic capability as ‘reprowebs’—an approach that adds a new dimension to the existing conceptualization of reproductive travel and helps us to better understand current developments in the global ART industry.

## Introduction

Aviad^1^ is an Israeli living in a large town in Germany with his German civil partner, Paul. A friend had once said to him that one should have children “when the wish is burning in your bones.” In the early 2010s, they felt this wish strongly and would have liked to adopt a child. This, however, was impossible in Germany at the time as same-sex couples were not yet allowed to adopt children who were not genetically related to either partner.^2^ The couple considered fostering a child, but were disheartened by the fact that foster children in Germany are often returned to their birth family when the situation there improves. Researching further options, they learned about gestational surrogacy—a medically assisted reproductive arrangement where a woman carries a genetically unrelated child for others. This alternative route to parenthood, however, would have been equally impossible in their home countries as Germany strictly prohibits all forms of surrogacy and Israel—which is generally surrogacy friendly—at the time still denied the right to surrogacy to gay couples.^3^ As a result, they finally chose to commission surrogacy elsewhere, namely in an unspecified location in India,^4^ using cryopreserved eggs donated by a South African woman, Aviad’s sperm, and an Indian gestational surrogate who was to carry the child. However, the surrogate did not become pregnant and before they were able to try again, India banned surrogacy for gay men. The couple decided to ship the left-over cryopreserved embryos from India to California, where preimplantation genetic screening found only one embryo to be chromosomally ‘normal.’ It was implanted into the uterus of a US surrogate; however, again, a pregnancy did not result. A subsequent try with a new egg donor from the U.S., the eggs again being fertilized with Aviad’s sperm, and two embryos subsequently transferred, was finally successful. Today, Aviad and Paul are fathers of twins (Interview with Aviad by Anika König, 22 May 2017).

This example illustrates the global routes, detours, and circumventions people such as Aviad and Paul, who are able to afford and make use of assisted reproductive technologies (ARTs), may take in order to become parents or expand their families. It also shows that these routes are strongly influenced by local laws (e.g., the prohibition or legality of certain practices) and the availability of technologies, knowledge, and reproductive workers in places Marcia Inhorn has termed ‘reprohubs’ (2015). While Germany and Israel did not allow for gay intended parents (IPs) to resort to gestational surrogacy, India did not only permit it, but several Indian cities used to be well-established and highly professionalized destinations for intended parents from abroad. Finally, the above example demonstrates that local changes and disruptions—especially the introduction of new laws—influence and alter the routes individuals are able to take, as happened with the prohibition of gestational surrogacy for same-sex intended parents in India which forced Aviad and Paul to turn to the United States to commission surrogacy there. But this example also shows how the reproductive industry operates as a whole as it requires a high level of mobility, flexibility, interconnectedness, and adjustment to disruptions: the closing of a reprohub may result in the opening of a new one, often just across the border (for example from India to Nepal and later to Cambodia), but also even on different continents (e.g., Indian clinics moving some of their activities to Kenya). In addition, especially in the last few years, ART arrangements have become more fragmented and hybridized, performing one step of the process (e.g., egg and sperm retrieval) in one location, and another (e.g., in vitro fertilization) elsewhere, only to be finalized (e.g., gestational surrogacy) in a different place again, as in Aviad’s and Paul’s arrangement with their South African egg donor, the Indian surrogate, the shipping of the embryos from India to the U.S., and the subsequent implantation of these embryos into the womb of a U.S. surrogate. All of these different locations are a part of, embedded in, and form global networks and structures.

In order to better understand how these increasingly complex global networks of people, substances, technology, money, and knowledge operate, are linked, interconnected, and continue to function—albeit in different ways—even in times of crisis (such as during the Covid-19 pandemic), in this paper we develop the approach of ‘reprowebs.’ This approach builds on, but extends, the well-established concepts of ‘reproscapes’ (Inhorn and Shrivastav 2010) and ‘reprohubs’ (Inhorn 2015) by adding a particular focus on the elasticity and changeability of the reproductive industry, its fragmentation, and the individual journeys people take within it. While the concept of ‘reproscapes’ is based on Arjun Appadurai’s approach to globalization (1996) and focuses on global flows and pathways of “people, technologies, finance, media, ideas, and gametes” (Inhorn and Shrivastav 2010, p. 68S), ‘reprohubs’ emphasize the importance of geographic locations where these people, technologies, finance, media, ideas, and gametes aggregate in order to provide reproductive services to clients from all over the world. The concept of reprowebs builds on both these concepts but lays additional emphasis on the elasticity of the global reproductive industry (i.e., its malleability to shift directions and expand into new markets and shrink from others) and its ability to flexibly react to change and disruption. Thus, similar to a spider’s web, reprowebs are global webs of reproduction which consist of nodes or hubs (i.e., reprohubs) which are dis/connected (Dilger and Mattes 2018) in various ways, rapidly react to change (such as locally changing laws or the global Covid-19 pandemic), and may alter in space and time. They are deeply embedded in globalization and their development is facilitated by conflicting national laws, new technological inventions, a globalized market economy, and the increased mobility of persons, substances, technology, knowledge, and money.

Our approach and the conceptualization of reprowebs is especially informed by our long-term ethnographic research on one particularly contested assisted reproductive technology: gestational surrogacy—an arrangement where a woman, a ‘gestational surrogate’ (sometimes called a ‘gestational carrier’), gestates an embryo she is genetically not related to and after birth hands the child over to the commissioning or ‘intended’ parents (IPs). While Heather Jacobson’s research focuses on gestational surrogates in the U.S., who carry children for U.S. and foreign IPs (Jacobson 2016, 2018, 2021), Anika König studies intended parents from Germany and Switzerland, who commission surrogacy outside their home country, especially in the U.S. and Ukraine (König 2018, 2020). Both authors have studied gestational surrogacy since 2009 (Jacobson) and 2013 (König), respectively, interviewed surrogates, IPs, agency personnel, and various other actors in the surrogacy industry and have closely followed and investigated the changes the reproductive industry has undergone since then, including the effects of the Covid-19 pandemic on reproductive travel and the reproductive industry (König et al. 2020). While our ethnographic data mainly originates in our surrogacy research, and the ethnographic data we use predominantly relates to this specific ART-enabled arrangement, the concept of reprowebs applies to all ARTs (most of which are also part and parcel of gestational surrogacy), such as in vitro fertilization (IVF), gamete donation, and preimplantation genetic diagnosis/screening (PGD/PGS) (also see Jacobson 2020).

We see reprowebs as one example among other global industries that take similar shapes and forms. The web analogy may be applied to other industries, especially those found in the realm of what has been termed ‘biocapital’ (Helmreich 2008; Sunder Rajan 2006, 2008; Vora 2015), such as the organ transplantation market and tissue economies (Scheper-Hughes 2000; Cooper and Waldby 2014), the medical travel industry (Vindrola-Padros 2020; Ormond and Lunt 2020), or, more broadly, global care and the “international division of reproductive labor” (Henshall, 1999; Boris and Parreñas 2010; Parreñas (2015). These global industries rely on connected webs of providers and adapt to shifts in the market and changing circumstances. As industries, they are also, of course, shaped and sustained by capitalism which drives the repro-industry just as it drives all industries participating in today’s globalized economy. However, while our notion of ‘webs’ is not exclusively applicable to the global reproductive industry, there are certain characteristics that specifically mark this industry and, therefore, make it unique and an interesting case for analysis. Most importantly, the fertility industry aims at the creation of human beings and thus combines a capitalist approach (which can be understood as the very essence of the various forms of biocapital) with what has often been described, building on Zelizer’s classic work (1985), as “precious yet priceless” (Berend 2015)—a child. From an economic perspective, and in contrast to most other industries, today, these precious children are “economically worthless” to their parents, which differs from previous centuries, places, and social strata where children contributed economically to their families (Zelizer 1985). Today, these children do not generate income but rather, create costs; while this is also already true for many families whose children were conceived ‘naturally’ and who do not depend on child labor, the fertility industry further increases the costs of these children by making their creation itself expensive. By making assisted reproductive services available and affordable for some, while excluding others who do not have the financial means, it is the industry itself (with its underlying logic of capitalism), that creates and maintains structural inequalities in the form of stratified reproduction (Colen 1995). Looking at different national and political approaches to reproductive technologies, we can also see how stratification may be increased or weakened, depending on the respective context. This can be seen, for example, in the different systems of funding, ranging from generous financial support for the use of ARTs, such as in pronatalist Israel (Birenbaum-Carmeli and Carmeli 2010), to many other countries, such as Mali (Hörbst and Wolf 2014), where ARTs are completely privatized and accessible only to those who can financially afford them (and who usually belong to socioeconomic strata whose reproduction is politically favored). As a result, within the realm of ARTs, reproductive inequalities do not only affect people differently within certain national contexts, but the commercialization of ARTs also creates stratification on a global level—between countries where most people can access them and others where all or most cannot. The creation and existence of “priceless” and “economically worthless” children (Zelizer 1985) thus cannot be divided from that which these children are often framed to be the opposite of: capitalism and commercialization.

As we will detail in this paper, this intersection of what Zelizer (1985, p. 11) frames as the “sacralization” of children, widespread cultural pronatalism, and marketized family building results in a robust globalized market with very ‘sticky’ connected webs of providers.

## The history of assisted reproductive technologies

The last five decades have been marked by unprecedented developments in the field of human reproduction. Hormonal contraceptives and treatments, in vitro fertilization, preimplantation and prenatal genetic testing/screening, and genome editing are just a few examples of medical inventions regarding reproduction that have emerged in the last decades. Many of these treatments and technologies originated as developments in the biosciences. As the resulting technologies moved from the lab to the clinic and began to shape people’s lives, they soon became subjects of interest to the social sciences. Especially since the beginning of the new millennium, a wealth of research has been published that analyzes the impact of new reproductive technologies on society and individual lives, on their politicization, commercialization, and location within global biopolitics (Whittaker 2015a). Research in this field ranges from New Kinship Studies (e.g., Carsten 2000; Edwards and Salazar 2008; Franklin and McKinnon 2000) to the study of particular ARTs (Almeling 2011; Inhorn 2015; Majumdar 2017; Rapp 2000; Van de Wiel 2020), and from transnational perspectives (Inhorn 2018; Knecht et al. 2012; König 2018; Nahman 2013; Speier 2016) to local and national research foci (Berend 2016; Bharadwaj 2016; Jacobson 2016; Teman 2010). Moreover, ARTs are studied from a variety of disciplinary backgrounds, including sociology (Almeling 2011; Franklin 2013; Hertz and Nelson 2018; Jacobson 2019), anthropology (Becker 2000; Birenbaum-Carmeli and Inhorn 2009; Edwards 2015; Ginsburg and Rapp 1995; König 2018; Majumdar 2017; Whittaker 2018), psychology (Ciccarelli and Beckman 2005; Golombok et al. 2013; Jadva et al. 2015), legal studies (Cahn 2009; Roberts 2009), and science and technology studies (Bhatia 2018; Murphy 2012; Thompson 2005).

If we take a look at the history of ARTs, Simpson and Hampshire (2015) have suggested that access to these technologies can be divided into three phases: the “first phase” spanning the 1980s and ‘90s when “extra-corporeal conception became available to a relatively small number of people in Europe, North America and the Middle East” at considerable financial costs. In the following “second phase”, ARTs became more widely available, now also being offered in countries all over the world, mostly by private providers (Simpson and Hampshire 2015, pp. 2–3). However, it was still a privilege of the wealthy to access these services, thereby reinforcing global and social inequalities. Subsequently, the second decade of the new millennium has seen the beginning of the “third phase” of medically assisted reproduction, a phase that is marked by “an extension of access and availability that further integrates ARTs into infertility treatment across the globe” (Simpson and Hampshire 2015, p. 4). In contrast to the previous two phases, ARTs have become “part of a standard repertoire of medical assistance for infertility” (Simpson and Hampshire 2015, p. 2), facilitated by the medicalization of infertility as a disease on the one hand (Greil et al. 2011; Conrad and Leiter 2004), and—in some locations such as the UK and Israel—the introduction of publicly funded infertility treatment on the other. In this third phase of ARTs, “this technology is geographically, ethnically, and socioeconomically open to ever-more constituencies” (Nahman 2016, p. 418).

One particularly salient feature of the fertility industry in the new millennium is that the movement of persons and gametes has considerably increased and, more often than previously, crosses national borders—a phenomenon that has been termed ‘cross-border reproductive care’ (Hudson et al. 2011; Inhorn and Gürtin 2011). This development brings with it challenges that usually do not affect those treatments which from beginning to end take place within one country. Rather, these challenges particularly concern arrangements taking place in different national settings, involving legal systems some of which may clash (Engel 2014; Kindregan and White 2013; Trimmings and Beaumont 2011), geographic distance (including different time zones that complicate communication and face-to-face interaction and require long-distance travel) (Ziv and Freund-Eschar 2014; Carone et al. 2017), and language barriers and cultural differences (Jacobson 2020), and as mentioned in many of Anika König’s interviews with intended parents from Germany and Switzerland). While reproductive treatments that span two or more countries are thus enabling on the one hand, they also complicate relations between the different actors involved in such treatments and thus have the potential for additional disruption. Although, as mentioned above, there are many similarities with other global industries, the deeply personal and emotional nature of creating a child in some ways thus makes this particular industry unique.

We argue that this third phase of assisted reproduction is the base on which reprowebs are built and the prerequisite for their existence: the global availability of ARTs to an ever-increasing number of persons, a high level of mobility of persons, substances, technology, and knowledge, world-spanning communication networks (e.g., the internet, email, and virtual meeting platforms such as Skype, WhatsApp, and Zoom), and a wide range of services of highly varying costs for these services. Reprowebs are possible due to the growth in size and scope of the global ART industry, and the increasing number of persons accessing it. The concept of reprowebs thus builds on the notion of this third phase of assisted reproductive technologies, with an emphasis on the issue of increased mobility and connectedness of patient/clients, but also of those who provide reproductive services, such as gamete donors and surrogates, as well as gametes, technology, and knowledge.

The mobility and connectedness of persons and things, which is a prerequisite for reprowebs, has been recently challenged with the Covid-19 pandemic, causing a sudden and severe disruption to the entire global reproductive industry by inhibiting travel of persons and, at least for a while, also of things (such as tissue). But this did not make reprowebs disappear—instead, one could say that the webs have tightened, contracted, and moved, making global connections more local (at least temporarily), offering alternative paths and connections (such as car travel instead of air travel or shipping cryopreserved gametes rather than collecting them in the destination clinic), or forcing a postponement of procedures and travel (such as intended parents not being able to pick up their children after birth or women having to delay medical procedures). We argue that this flexibility regarding the change of external circumstances is an integral characteristic of reprowebs, therefore allowing us to analytically grasp changes in space and time. We may even speak of a ‘fourth phase’ of reproduction now, as the pandemic may be causing lasting change to the global ART industry. This, of course, is an empirical question, but one we posit worthy of investigation.

## Reproductive travel and reproductive hubs

Underlying the idea of reprowebs is the fact that large numbers of people who have the financial means to afford ARTs do not, or not exclusively, access reproductive services in their immediate locales and often not even in their home countries. Instead, they travel elsewhere to access these services–they become what we, following Inhorn (2015), call ‘reproductive travellers’ or ‘reprotravellers’. Other terms for this particular kind of medical travel are ‘reproductive tourism’ (Ikemoto 2009; Nahman 2016; Pennings 2002), ‘fertility tourism’ (Bergmann 2011a), ‘transnational reproduction’ (Deomampo 2016), ‘cross-border reproductive travel’ (Whittaker and Speier 2010), ‘reproductive trafficking’ (Franklin 2012), ‘reproductive exile’ (Inhorn and Patrizio 2009), or the above-mentioned ‘cross-border reproductive care’ (Hudson et al. 2011; Inhorn and Gürtin 2011), all of which have been subject to debate (Inhorn 2011). While ‘reproductive’ or ‘fertility tourism’ has been criticized for its insinuation of pleasure (Casper 2011; Hudson et al. 2011, p. 677) which is in stark contrast to the experiences of many people who access reproductive services away from home, the other terms aim at taking a less biased stance. We have chosen to use ‘reprotravel’ as it emphasizes mobility but can also include travel within a country as well as between different regions or states.

The reasons for reproductive travel vary widely, from the lack of availability of certain technologies or their unlawfulness in the reprotravellers’ home country or town (Frankfurth 2020; König 2018, 2020), to financial considerations when certain treatments are cheaper elsewhere (Speier 2016), the desire to conceal the treatment from neighbors, friends, or family or to undergo medical treatment near family in one’s native ‘home country’ (Inhorn 2018), to the “relatively quick and procedurally smooth access to controversial and complex ART services” available in only certain national markets (Jacobson 2020, p. 43, also see Martin 2015).

Reprotravel is a prerequisite for the emergence of ‘reprohubs’ (Inhorn 2015) which are geographic places or centers where certain factors come together, making these places particularly attractive to reprotravellers: they offer medical treatments such as in vitro fertilization (IVF), intracytoplasmic sperm injection (ICSI), surgery and/or hormonal treatments; in many cases there are also gamete donors/banks and gestational surrogates available in these places; there are agencies that broker contact between those who want to access gametes or surrogacy services and the providers of these services (i.e., gamete donors and surrogates); there are the laboratories and embryologists, who can provide additional services like preimplantation genetic diagnosis (PGD) and preimplantation genetic screening (PGS); the local and/or national legal system permits the employment of these assisted reproductive technologies; local attorneys provide legal services; there is an infrastructure that allows for easy travel to and from these places; and local agency and medical staff speak global languages such as English (or employ interpreters) in order to cater to foreign clients. In short, reprohubs are places where reproduction is “territorialized in assemblages” (Ong and Collier 2007 [2005], p. 4).

In her book *Cosmopolitan Conceptions* (2015) Inhorn shows how Dubai has become such a reprohub: it has a suitable infrastructure and geographical location, as it is a popular tourist destination which offers special visas and, “[m]oreover, the UAE is located in the geographic center of many other Muslim nations, serving as a kind of hub for the Muslim diaspora” (2015, p. 2). Inhorn also shows that the United Arab Emirates “represents a zone of cultural comfort in a country that undertakes IVF according to Islamic Sharia laws” (2015, p. 2) since “Muslim clerics there had legitimized IVF to overcome infertility many years earlier” (2015, p. 9). Other reprohubs have been located all over the globe, from the Czech Republic (Speier 2016) and Spain (Bergmann 2011b) to the U.S. (Jacobson 2018, 2020; Martin 2015) [especially California (König 2018)], Russia (Brednikova et al. 2009; Weis 2015), Ukraine (Siegl 2018), and Mexico (Hovav 2019).

Until recently, India was one of the most important reprohubs worldwide (Bharadwaj 2016; Deomampo 2016; Majumdar 2017; Pande 2014; Rudrappa 2015; Saravanan 2018), followed by Thailand (Whittaker 2015b) on a smaller scale. Both countries, however, recently banned one of the  arrangements they were perhaps best known for: transnational commercial gestational surrogacy. In India, the law was changed as a result of political pressure, first banning surrogacy travel into India in 2012 for homosexual intended parents, and in 2016 for all foreigners (Rudrappa 2018b). Thailand was struck by several scandals (Whittaker 2016), especially the case of ‘Baby Gammy’, a child with Down Syndrome, whose commissioning parents allegedly left him in Thailand while taking Gammy’s twin sister, who was not affected by the condition, home with them. Almost at the same time, the ‘baby factory case’ drew international attention when a Japanese businessman reportedly commissioned multiple gestational surrogacies simultaneously, resulting in the birth of 13 children within just a few weeks/months  (Horton 2018). Thailand reacted quickly and, like India, banned surrogacy for foreign intended parents (Whittaker 2018).

Inhorn argues that “the emergence of such […] reprohubs is a new and unique by-product of twenty-first century reproductive mobilities” (2015, p. 9). While this is certainly still true for many places that attract reprotravellers, with our concept of ‘reprowebs’ we want to extend the conceptualization. The emergence of reprowebs is a result of the recent developments in places like India or Thailand which have banned surrogacy or which, like Ukraine, only allow the use of certain ARTs for certain clients, excluding, for example, same-sex couples or single intended parents. Clinics, agencies, and clients have found ways to circumvent these local restrictions by outsourcing some, or sometimes most, elements of the ARTs they offer to third locations, a strategy that has also been called a ‘hybrid’ model for surrogacy arrangements (Whittaker 2018, p. 175).

One example demonstrating the concept of reprowebs was found in an interview (by Anika König) in 2019 regarding gestational surrogacy for same-sex couples in Ukraine. A surrogacy agent in Kiev revealed that while the agency and clinic are based in Ukraine and the gestational surrogate herself is Ukrainian, the surrogate travels across national borders and undergoes the necessary medical treatments elsewhere and/or gives birth in a country whose laws are ‘friendlier’ regarding same-sex intended parents. In interviews with surrogates in 2009, Heather Jacobson learned of similar circumventions of local regulations within the U.S., whereby agencies would contract with surrogates in one state yet have them travel across the border to another to give birth in a legally more ‘surrogacy friendly’ state. Likewise, after the Indian ban of surrogacy for homosexual intended parents, Indian agencies and clinics offered the option of sending cryopreserved sperm to India where it was used to create embryos with Indian donor eggs which were subsequently implanted into Indian surrogate mothers’ wombs. Some Delhi ART clinics, which already had established networks with clinics in legally less restrictive Nepal, brought the pregnant surrogates to Kathmandu where they gave birth and the commissioning IPs could pick up the child (Rudrappa 2018b, p. 81). Indian clinics from Mumbai expanded to Kenya, where they recruited local women to act as surrogates, flew them to South Asia for treatment, and later flew them back to Kenya to give birth there (Rudrappa 2018b, pp. 81–82). Similarly, a U.S. surrogacy agent who arranges such ‘hybrid’ surrogacies, told Anika König that Indian egg donors are flown to the country of Georgia in order to undergo stimulation and egg harvesting there, while Georgian surrogates who are carrying children for same-sex intended parents (which is prohibited in Georgia) are flown to Mexico for treatment and delivery of the child (interview with Ruby 6 May, 2021).

Andrea Whittaker (2018, p. 175) describes “this form of surrogacy [as] FIFO (fly in fly out)—a term used in Australia to describe workers in extractive industries such as mining who regularly fly into their remote workplaces, and fly out again to return home at the end of their fortnight’s work.” Similar strategies have been reported with regard to Thai doctors, who after the Thai ban in 2015 relocated to Laos where they treated Thai surrogates and delivered the babies they carried to be picked up by the commissioning parents there (Rudrappa 2017).

Accordingly, many ART treatments and arrangements, from beginning to end, do not take place exclusively in one reprohub anymore, but involve people, biological substances, and reproductive and legal services from a range of different places, thereby circumventing local restrictions and laws, beating down prices, and disguising business activities. Whittaker (2018, p. 176) even argues that

[a]s “one-stop” [i.e., reprohub] surrogacy countries have become increasingly rare and legally fraught, the creative fragmentation of the supply chain into a number of different stages, each carried out within different jurisdictions, has become the norm.

The webs of travel, treatment of people, and movement of substances that now often cover several regions, countries, and sometimes even continents, is what we call reprowebs. Accordingly, rather than disappearing as a result of new legal restrictions or travel limitations, reprohubs extend and transform into integrated reprowebs, becoming more flexible, fluid, and elastic, thereby better adapting to changing circumstances.

## Networks, meshwork, and reprowebs

We have chosen the term ‘reproweb’ to articulate and analyze the above-described recent developments in the global reproductive industry. Our concept is based on the particular features of a spider’s web which stand in contrast to other kinds of networks in that it develops organically, is strong and flexible, and does not follow a preparatory plan.

This idea was further elaborated by Tim Ingold (1997, p. 109) who argues that in contrast to a fisherman’s net, for example, there is no ‘design agent’ in the construction of a spider’s web, and that “[t]he web, in short, is the product of behavior that is genetically programmed and that is carried out, to all intents and purposes, without any conscious direction or intentionality.” Accordingly, a spider’s web organically adapts to the situation within which it is created, rather than being planned and then realized according to the plan. This, however, does not mean that actors within the reproductive industry have no plan or idea where they would like to move their enterprise or with which other clinics or providers they would like to connect their practice. Rather, reprowebs depend on the established networks created by providers, honed in interactions and exchanges between agencies, clinics, and attorneys.

As social network theorists have argued, strong ties between people, built overtime in interaction, create strong networks (Pescosolido 2007). Within the ART industry, as within most industries, organizations, and structures, there is variability in terms of the strength of ties between stakeholders. When new conditions arise, resulting in changes to the local environment which cannot be determined beforehand, classical preparatory plans are unlikely to work. However, strong networks—which, for ART clinics and practitioners, take time, effort, money, and social and cultural capital to build—often enable the continuation of organizations within such circumstances. A good example of this, as we will discuss at more length, is the Covid-19 pandemic in which the changes wrought to the global ART industry could not have been predicted or planned, yet the flexibility of reprowebs enabled the market to quickly adapt.

Elsewhere, borrowing from Lefebvre’s work on *The Production of Space* (1991 [1974]), Ingold develops the concept of ‘meshwork’ which bears some similarity with our notion of webs and clearly differs from networks: while “[t]he lines of a network, in its contemporary sense, join the dots” (Ingold 2007, p. 80), the lines within a meshwork “are the trails along which life is lived” (Ingold 2007, p. 81). Thus, the emphasis is on the “entanglement of lines, not in the connecting of points” (Ingold 2007, p. 81). However, our concept of reprowebs differs from meshworks in one important aspect: Ingold opposes the connecting of points (i.e., a network) to the entanglements of a meshwork. Reprowebs, in contrast, can be understood as a combination of the two, networks and meshworks, the connection of dots on the one hand, and an entanglement of paths on the other, thereby forming a third kind of structure: a web. This becomes clear when we revisit our introductory example of Aviad and Paul.

In Aviad and Paul’s case we deal with the connection of dots (i.e., reprohubs) in South Africa, India, and California through travel and communication. But the routes between these and other places are entangled, frequently change direction, influence one another, and have meaning in themselves. A change in India altered the couple’s direction toward the U.S., several disruptions in the U.S. led to new directions in the routes taken there, and so on. While some of these routes connecting the different reprohubs formed a network, the entanglements formed a tissue or meshwork “which is continually being woven as life goes on along them” (Ingold 2007, p. 84), and in this very particular combination the two constitute what we call a reproweb. Moreover, these reprowebs do not constitute bounded entities, simply surrounded by an environment. Rather, the environment can be understood as a zone within which the web continually moves, changes, and expands as, similar to a meshwork, “its ever-extending lines probe every crack or crevice that might potentially afford growth and movement” (Ingold 2007, p. 103).

If we take a closer look at the developments the global reproductive industry has undergone in the last decade or so, the probing of cracks and the growth into crevices become evident: Kazakhstan and Kenya, for example, constitute such new niches (Guseva and Lokshin 2019; Rudrappa 2018a), as they have become new destinations for surrogacy travel or for at least some stages of fragmented surrogacy arrangements within reprowebs. While Kenyan clinics and doctors had been in contact and collaborating with Indian fertility professionals (as mentioned above), the Kazakh fertility industry had been collaborating with Russian and Ukrainian fertility doctors for a long time (Guseva and Lokshin 2019) which is why this particular ‘crack’ or ‘crevice’ was by no means incidental. In the German-speaking region, since the legalization of egg donation in 2015 Austrian clinics are advertising for treatment, specifically targeting German-speaking clients from the two neighboring countries Germany and Switzerland who seek access to IVF treatment with donor eggs, but whose home countries prohibit egg donation. Other cracks, like India, have closed, causing yet another change of direction of route in reprowebs, both for the industry as a whole and for individuals seeking treatment. Accordingly, the environment (or zone) within which the industry operates is subject to constant change, such as new legal regulations (e.g., the prohibition of surrogacy in India, Thailand, and Nepal), the opening up of new locations (e.g., Kazakhstan, Kenya), and the introduction of new technologies (e.g., the cryopreservation of oocytes which makes it possible for them to be shipped around the globe).

Although reprohubs continue to exist, they bleed out to other locations, making their services multi-local, constantly adapting to new obstacles and opportunities. Consequentially, reprowebs do not only describe a particular moment in time, but also, and importantly, reprowebs have a process-related quality concerning the ways they build up, develop, and change over time. This is not to say, of course, that these are spider-less webs, created without actors. Taking Nancy Krieger’s (1994) critique of the use of the web analogy in epidemiology to heart, we understand reprowebs to be a response by the global ART fertility industry, composed of multiple kinds of stakeholders who have often worked to establish connections with each other, to constant change. Accordingly, the actors within this web are at the same time integral parts of it.

Reprowebs, as a particular combination of networks and meshwork, also capture the feelings of entrapment some ART patient/clients experience in fertility treatment. Similar to a fly trapped in a spider’s web, some ART clients feel unable to remove themselves from treatment due to the promise that these ‘hope technologies’ hold (Becker 2000; Franklin 1997; p. 33, Jacobson 2016; Speier 2016). This ‘commitment’ to ART treatment is not new. Margarete Sandelowski (1991, p. 30) already noted nearly three decades ago:

Clinicians, social scientists, journalists, and social critics of conceptive technology have described couples as “driven” in their pursuit of pregnancy, as feeling that they have no choice but to undergo certain treatments﻿—even as being addicted to treatment (Frank 1989; Greil and Porter 1988; Olshansky 1988).

Sarah Franklin (1997, p. 11) has shown that the use of ARTs can even become a “way of life” as they “take over.” The next round of treatment may be a success, and especially when a new option becomes available, such as a new technology or a new clinic nearby, women in her study claimed that they felt they “had to try” (Franklin 1997, p. 170), even if that resulted in tremendous cost and effort. As Marcia Inhorn (2018, p. 148) articulated, “when people are desperate to have a child, they will often undertake difficult border crossings—valiant quests for conception that are costly, time consuming, and physically and emotionally arduous.” We posit that the vocabulary of a reproweb captures well that feeling of ART treatment entrapment for as Jiménez (2018, p. 53) has noted in his analysis of usefulness of the web as trap analogy, “the spider-web-trap is an ecology, but it is also an entanglement, and it is also an infrastructure.”

We further propose that the nature of the ART industry today, seemingly able to adapt to legal restrictions, social mores, or logistical hurdles via reprowebs, provides continual possibilities for a more expanded patient/client base’s sustained pursuit of treatment in ways previously unavailable. As one treatment failure leads to the next, legal restrictions appear, or the introduction of various technologies (like preimplantation genetic screening) suggest new directions, global reprowebs are under constant pressure and subject to disruptions, but at the same time they possess a unique ability to adapt and allow not only for individual patient/clients to remain in treatment—as long as they can afford it—but for the ART industry to remain viable.

The ability of reprowebs to nimbly adapt to change is inpart due to the interconnectedness of those in the industry. Just as spiders use their instinctual knowledge about their environment and engage in repeated effort to build, rebuild, and adapt their webs (which enable prey to be trapped, thus feeding them) to their environments, stakeholders in the ART industry (especially successful ones) come to understand their working environments and the particular local politics that may shape their practices. Like the spider, surrogacy stakeholders labor to build webs which in this case consist of networks of collaborators and vendors that allow for the creation of professional connections, that in turn create structures that ‘feed’ the industry with their seemingly endless supply of enticed patient/clients. Reprowebs are also not random: in the same way spiders do not randomly build webs, reprowebs rely on a variety of external factors, such as stakeholders’ inside knowledge of environments, politics, other actors, and industry patterns and the connections established between working practitioners via their interactions.

Though these reprowebs enable the industry to survive, the flipside of the increased complexity, fragmentation, and hybridization resulting from these webs amplifies the vulnerability of the persons involved in multi-local ART arrangements—most importantly surrogates and egg donors, but also intended parents and children (Whittaker 2018), especially concerning legal certainty. A good example of this was the response to the 2015 earthquake in Nepal, part of a reproweb connecting three countries: India, Nepal, and Israel. Indian surrogates pregnant for Israeli intended parents were trapped in Nepal following the earthquake. The surrogates had been moved there to evade the prohibition of surrogacy for foreign intended parents in India. These women were carrying for Israeli gay male couples, as Israeli surrogacy laws do not allow for same-sex male couples to commission Israeli surrogates. The internationally operating surrogacy agency was located in Israel; with their local partners they recruited Indian nationals who spent their pregnancies in Kathmandu as Nepal did not allow for Nepali women to act as surrogates (Shalev et al. 2017). Following the earthquake, the Israeli government responded quickly, flying out Israeli intended parents and their 26 children born via surrogacy on governmental transport, while leaving behind the surrogates, including those pregnant for other Israeli intended parents. Following worldwide publicity and outrage, those surrogates still pregnant with Israeli babies were flown to Israel to complete their pregnancies and give birth there (Eglash 2015; Rudrappa 2018a; Shalev et al. 2017).

As demonstrated with this Nepalese example, conflicting legal regulations and the global fragmentation and hybridization of treatments make it hard or impossible for reproductive workers such as surrogates or egg donors to seek legal help when there is a problem; in the case of transnational gestational surrogacy, intended parents often underestimate the ‘document work’ they have to invest into legally becoming their child’s parents if the laws of the birth and the home country regulate this differently; and there have been several cases of children remaining stateless for months or years because their birth country regards them as citizens of their intended parents’ home country, while the latter sees them as citizens of the former (Ergas 2013; Kindregan and White 2013; Smerdon 2012). As we show below, these vulnerabilities became particularly salient during the Covid-19 pandemic.

## Pandemic contractions and disruptions

The Covid-19 pandemic caused major disruptions to the previously steadily growing global fertility industry. Professional associations such as the European Society of Human Reproduction and Embryology (ESHRE) and the American Society for Reproductive Medicine (ASRM) “in the earliest stages of the pandemic […] recommended discontinuation of reproductive care except for the most urgent cases” (Veiga et al. 2020, p. 1). On the one hand, fertility specialists worried that an infection with the SARS-Cov-2 virus or medication used in the case of an infection could have a negative impact on the pregnancy, and on the other, the overstrained healthcare systems should not be burdened with treatments that could be postponed to a later point in time [European Society of Human Reproduction (ESHRE) 2020]. As a result, in many countries, ART treatments were halted or postponed, and entire fertility clinics closed. While reports abound about persons who were seriously affected by these changes in the fertility treatment sector, the ‘pandemic disruptions’ (König et al. 2020) especially hit persons receiving treatment that involved third persons and/or for which national borders had to be crossed, as is the case with transnational surrogacy.

For example, in May 2020, news reports described the fate of children born through surrogacy in Ukraine, who could not be picked up by their intended parents because of closed borders and travel bans (Harley-Mckeown 2020; Kramer 2020). Photos and videos showed around 50 screaming infants in several rows of cribs in a large hotel hall in Kiev taken care of by nurses and nannies (e.g., Berdnyk and Goncharenko 2020). The original video^5^ had been posted and spread by the largest fertility clinic in Ukraine, BioTexCom (which possesses a large and active PR department), trying to draw attention to the problem of children being stuck in Ukraine. The text accompanying the video urged intended parents to “take action.” At the time, foreigners needed a special permit to enter Ukraine, which they could only receive with the help of their embassies or the Ukrainian human rights commissioner’s office. Accordingly, the video is likely to have been disseminated to pressure foreign governments to support their citizens in acquiring entry permits to Ukraine in order to be able to collect their children. The video caused outrage both in Ukraine and around the world, fueling existing criticism of surrogacy, especially in ‘low-cost destinations’ like Ukraine (Grytsenko 2020; Vlasenko 2020). In international news reports, the Ukrainian ombudsman for human rights and outspoken critic of surrogacy, Lyudmila Denisova, was said to “propose[…] to change the laws in order to allow only Ukrainians use such services” as “[c]hildren in Ukraine must not be subject to human trafficking” (Ilyushina 2020). In contrast to the swift reaction of the Thai government in the cases of Baby Gammy and the Baby Factory (which led to a rapid prohibition of surrogacy arrangements for foreign commissioning parents and commercial surrogacy in general), to date Ukraine has not taken action to prohibit surrogacy. While the cracks closed in Thailand (as well as India and other countries) and the reproweb thus stretched and relocated, in Ukraine it just became a web under pressure—pressure it was able to endure. It did so by contracting part of the web: for a portion of 2020, Ukrainian surrogacy agencies refused to work with intended parents from certain countries, as clearly stated on their websites. The reproweb was sustained, however, as this restriction did not apply to clients from countries from where Ukraine could be reached by car, such as Austria or Germany. Ukraine continues to be a popular destination for reprotravellers seeking surrogacy and other ART services such as egg donation or IVF.

A similar development was noted in the U.S., where several news reports portrayed surrogates who had agreed to look after the children they had birthed until their parents would be able to enter the country—sometimes for up to several months (Dodge 2020; Kale 2020). This situation troubled surrogacy agencies, one of which reported to Heather Jacobson in an interview completed in May 2020 that they were contemplating restricting their practice to surrogates and IPs who resided not only within the U.S. but within the same state. This was due to state-level travel restrictions and quarantine requirements and hospital-specific Covid policies which dramatically changed the normal procedures of surrogacy. In this U.S. example as well as the case of Ukrainian agencies above, the reproweb contracted as a reaction to the Covid-19 pandemic and crisis, valuing proximity and preventing ‘long-distance contracts.’

More recently, additional alternative paths have been established within global reprowebs to supersede international travel, especially in the context of collecting gametes. While prior to the pandemic, “initial workups—including blood tests, hormonal screenings, and ultrasounds” of international intended parents would sometimes be completed by clinics in their home countries to cut down on the cost of travel to the U.S. and donor gametes would often be cryopreserved and shipped within a country or even transnationally,^6^ intended parents’ own sperm and oocytes were usually collected onsite in the fertility clinic that performed the IVF and embryo transfer (Martin 2015, p. 102). This especially concerned arrangements which included third persons (such as in surrogacy), as tissue used for these kinds of reproductive treatments must usually undergo tests and screenings in FDA (Food and Drug Administration)-approved laboratories in the U.S. (Practice Committee of the American Society for Reproductive Medicine and the Practice Committee for the Society for Assisted Reproductive Technology 2021). For intended parents this meant that they had to travel to their fertility clinic, even if it was abroad or across the country. After the introduction of travel restrictions during the Covid-19 pandemic, such procedures were not possible. While in the early months of the pandemic, a solution to this problem was limiting access to these ARTs to people living in the same country, state, or region, clinics catering to an international clientele have found ways to replace the travel of persons with the travel of tissue (e.g., blood samples and cryopreserved gametes). In the U.S., this poses a particular problem since, as mentioned above, donor and donor tissue are required by the FDA to be tested in approved laboratories.

Prior to the pandemic, gamete donor and donor tissue testing were usually completed in-house, at the U.S. fertility clinic. Today, several U.S. clinics have partnered with clinics abroad which collect blood samples and gametes for them by using certified FDA kits. These kits include a specimen bag with several containers, a requisition form, and detailed descriptions for the lab.^7^ An Italian intended gay father living in Switzerland (interview by Anika König, 17 February 2021) recounted that the Florida fertility clinic he had commissioned for his egg donation and surrogacy had ordered such an FDA kit to be shipped to his house in Switzerland. With this kit, he traveled to a clinic in Austria that cooperated with his U.S. clinic, had his blood drawn, and gave a sperm sample. The Austrian clinic first sent his blood sample to the lab in Fort Lauderdale, Florida, and only after the tests there did not show any abnormalities did the Austrian clinic ship the cryopreserved sperm for further testing. The tested sperm was then sent to the U.S. fertility clinic where the donor eggs were fertilized and transferred into the surrogate’s womb. With this procedure, it is possible to initiate a surrogacy arrangement without ever having traveled to the U.S. However, as several European countries prohibit egg donation, similar procedures cannot be done in those countries with oocytes. In such cases, intended parents (interview with intended father by Anika König, 3 March 2021) have been offered a medical certificate issued by their U.S. fertility clinic which can be used to acquire a travel permit into the U.S. for the person whose egg are harvested.

These new paths and threads within reprowebs do not only emerge with regard to ART treatment during the pandemic—they are also created when it comes to the birth of the children; this could be seen when intended parents were unable to collect their children due to travel restrictions. One case of this in our interview data was seen for a German mother, whose child was born through surrogacy in the U.S. in May 2020. She was unable to enter the country before her daughter’s birth; however, she could do so immediately postpartum as she was then the mother of a U.S. citizen under the age of 21 (interview by Anika König, 15 December 2020). Other intended parents, especially those whose children were born later in 2020, benefitted from the option of a ‘national interest exception.’ On the American Bar Association’s website (Vaughn 2020), we learn that

[t]he basis of this exception is that it is in the national interest of the United States that the intended parent(s) be allowed to travel to the U.S. to be present for the birth of the child so that they can make medical decisions for the child and take the child from the hospital as soon [as] possible… preserving U.S. healthcare personnel and resources for medical emergencies, including the ongoing emergency of the pandemic.

As our interviews with international intended parents and U.S. agencies and clinics have shown, an increasing number of international IPs is making use of this option to evade U.S. travel restrictions. Finally, European intended parents have also been instructed to travel to countries which are not affected by the U.S. travel ban (such as Mexico), quarantine there for two weeks, and then from those locations enter the U.S.—advice for international IPs that is also given on the American Bar Association’s website (Vaughn 2020).

These examples from Europe and the U.S. illustrate that even and especially in a time of crisis, reprowebs reveal their strength and flexibility: instead of disappearing, they adapt to the new situation, relocating, contracting, and being sustained by improvisation in an unprecedented global crisis: whether it was the Ukrainian clinic that produced and disseminated the video of stranded newborns in Kiev, the surrogates in the U.S. who agreed to take care of the children they had carried and birthed, the embassies that supported their country’s citizens in the acquisition of special travel and entry permits, lawyers who applied for national interest exceptions or showed international clients the legal loopholes for evading travel restrictions, the agency personnel who took photos and videos of newborns in order to be sent to their parents, or the countless nannies and nurses who took care of temporarily parentless children night and day. All of them and many more, human and non-human actors, threads, and their entanglements make up, sustain, and drive the global reprowebs we have described in this paper.

## Conclusion

The concept of reprowebs, proposed in this paper, allows for a more expansive conceptualization of the global reproductive care market. Reprowebs capture the new elasticity of the industry as it adapts to changing national legislation, the opening, closing, and development of local markets, and the travel restrictions and new institutional regulations resulting from the global Covid-19 pandemic. We offer this concept as an accompaniment to the existing conceptualizations of reproductive travel and reprohubs and argue this approach helps to better understand the current dynamic development of the contemporary global ART industry. With some small modifications, it could be adapted to other industries (e.g., as ‘biowebs’). Reprowebs also provides a vocabulary for us to explore the developments within the reproductive industry since early 2020, which are marked by unprecedented disruptions, but also alternative paths and strategies.

We posit that the concept of reprowebs can serve as a lens through which we can investigate change, both in space and time, in reproductive markets, as well as the recent fragmentation and hybridization of the industry which has not yet received much attention. The theoretical framework of reprowebs, like a spider’s web, allows for the mapping and analysis of the ways in which ART markets are spun, built on networks of professional relationships, reactive to change, and adaptive to problems that arise. Reprowebs capture how the global ART industry is facilitated by globalization, the conflicting national laws, new technological inventions, a globalized market economy, and the increased mobility of persons, substances, technology, knowledge, and money.

## Footnotes

1 In order to protect research participants’ identities, all names are pseudonyms and other identifying information was changed.

2 With the introduction of the so-called ‘Ehe für alle’ (marriage for everyone) in 2017, it has become possible for homosexual couples to adopt children who are not genetically related to either of the partners.

3 In February 2020, Israel’s High Court ruled that the country’s surrogacy laws which prevent gay and single men from accessing surrogacy within Israel “disproportionately violate the right to equality and the right to parenthood of these groups and are illegal.” The Knesset, however, did not succeed in changing the law within the given one year (later extended to one and a half years) and in July 2021, the High Court ruled that the “all legislation denying surrogacy rights to same-sex couples and single men will be null and void six months from now “ (Bachner 2021).

4 It is interesting that most of Anika König’s research participants who commissioned surrogacy in India, did not specify the exact location within India of the agency with whom they contracted nor where their surrogate was located. In contrast, those whose surrogacy took place in the U.S. usually mentioned at least the state where their surrogacy took place, and often also the town. This is certainly linked to the fact that many Germans are more familiar with U.S. geography than with that of India, but it is also symbolic of global hierarchies and colonial imagery within which countries of the Global South are not only less known, but also seem undifferentiated, incomprehensible, and exotic.

5 Online: https://youtu.be/xPdRx_L96C0 (Accessed 26 Jan 2021).

6 Though “frozen cycles” have increased in popularity, some clinics or intended parents continue to prefer so-called “fresh cycles” in which donor eggs are harvested and then fertilized without prior cryopreservation. Usually, the resulting embryos are subsequently transferred into the carrying woman’s womb. Fresh cycles, however, require the synchronization of donor and carrying woman’s cycles and both need to come to the same place for egg harvesting and embryo implantation (Jacobson 2019).

7 See, for example: https://www.infertilitylab.com/fda-donor-screen-handling-prep (Accessed 10 Mar 2021).
